# Integrative Multi-Omics Approach in Vascular Ehlers–Danlos Syndrome: Further Insights into the Disease Mechanisms by Proteomic Analysis of Patient Dermal Fibroblasts

**DOI:** 10.3390/biomedicines12122749

**Published:** 2024-11-30

**Authors:** Nicola Chiarelli, Valeria Cinquina, Nicoletta Zoppi, Valeria Bertini, Marianna Maddaluno, Chiara De Leonibus, Carmine Settembre, Marina Venturini, Marina Colombi, Marco Ritelli

**Affiliations:** 1 Division of Biology and Genetics, Department of Molecular and Translational Medicine, University of Brescia, 25123 Brescia, Italy; valeria.cinquina1@unibs.it (V.C.); nicoletta.zoppi@unibs.it (N.Z.); valeria.bertini@unibs.it (V.B.); marina.colombi@unibs.it (M.C.); 2 Telethon Institute of Genetics and Medicine (TIGEM), 80078 Pozzuoli, Italy; m.maddaluno@tigem.it (M.M.); c.deleonibus@tigem.it (C.D.L.); settembre@tigem.it (C.S.); 3 Department of Clinical Medicine and Surgery, Federico II University, 80138 Naples, Italy; 4 Division of Dermatology, Department of Clinical and Experimental Sciences, ASST Spedali Civili and University of Brescia, 25123 Brescia, Italy; marina.venturini@unibs.it

**Keywords:** extracellular matrix, collagen type I, collagen type V, miR-29b, multi-omics approach, proteome, transcriptome, vascular Ehlers–Danlos syndrome

## Abstract

**Background**: Dominant mutations in *COL3A1* are known to cause vascular Ehlers–Danlos syndrome (vEDS) by impairing extracellular matrix (ECM) homeostasis. This disruption leads to the fragility of soft connective tissues and a significantly increased risk of life-threatening arterial and organ ruptures. Currently, treatments for vEDS are primarily symptomatic, largely due to a limited understanding of its underlying pathobiology and molecular mechanisms. **Methods**: In this study, we conducted a comprehensive analysis of the intracellular proteome of vEDS fibroblasts, integrating these findings with our previous transcriptome results to identify key molecular pathways that drive the disease. Additionally, we explored the therapeutic potential of inhibiting miR-29b-3p as a proof of concept. **Results**: Our integrative multi-omics analysis revealed complex pathological networks, emphasizing the critical role of miRNAs, particularly miR-29b-3p, in impairing ECM organization, autophagy, and cellular stress responses, all of which contribute to the pathogenesis of vEDS. Notably, the inhibition of miR-29b-3p in vEDS fibroblasts resulted in the upregulation of several differentially expressed target genes involved in these critical processes, as well as increased protein expression of essential ECM components, such as collagen types V and I. These changes suggest potential therapeutic benefits aimed at improving ECM integrity and restoring intracellular homeostasis. **Conclusions**: Overall, our findings advance our understanding of the complex biological mechanisms driving vEDS and lay a solid foundation for future research focused on developing targeted and effective treatment strategies for this life-threatening disorder.

## 1. Introduction

Vascular Ehlers–Danlos syndrome (vEDS), considered one of the most severe subtypes of EDS [1], is caused by dominant mutations in the *COL3A1* gene, which encodes the α1 chain of type III collagen (COLLIII). COLLIII is the major collagen in blood vessels and hollow organs, playing a crucial role in conferring strength and flexibility to these structures [2,3]. A deficiency in COLLIII leads to soft connective tissues fragility, resulting in increased bruising and heightened risk of arterial and bowel ruptures, as well as fragility in the uterus, cervix, and vagina during pregnancy and delivery [4,5,6,7,8,9,10,11,12].

The diagnosis of vEDS involves evaluating major and minor clinical criteria defined in 2017, alongside confirming the presence of a pathogenetic *COL3A1* variant through molecular testing [13]. Ruptures of hollow organs, such as arteries, uterus, and intestines, can unpredictably occur in youth, with arterial ruptures being the foremost cause of mortality [4,5,6,7,8,9,14,15]. Currently, there are no approved therapies for treating vEDS patients, and the outcomes of endovascular and surgical treatment are poor, making prevention essential [16]. The Beta Blocker in Ehlers–Danlos Syndrome Trial (BBEST), conducted in 2010, reported a 64% decrease in the risk of arterial rupture or dissection in patients receiving celiprolol compared to controls [17]. Celiprolol, a β1 cardio-selective blocker with a β2-agonist vasodilator effect, is widely used off-label in Europe. However, its actual therapeutic benefits, underlying mechanisms of action, and overall efficacy remain poorly understood and subject to ongoing debate. Indeed, recent findings indicate that despite celiprolol therapy, vEDS patients still face significant burden of serious vascular complications [18]. Furthermore, after clinical trials in the USA [19], celiprolol was declined FDA approval due to poor tolerance in one-third of patients. Since the BBEST trial, no other medication has been definitely shown to prevent vascular complications in vEDS patients; however, ongoing trials are exploring other cardiovascular drugs, including angiotensin receptor blockers, which have shown promising results in mouse models [20]. Additionally, targeting specific signaling pathways such as PKC/MEK/ERK (e.g., with cobimetinib, a MEK inhibitor) has been shown to improve survival in some but not all *Col3a1* mouse models [21]. Considering the multisystemic nature of vEDS affecting diverse cell types, the effectiveness of targeting individual downstream pathways may be limited to specific cell types and/or mutations, as observed with celiprolol treatment in mice [20]. This underscores the need for new treatment strategies directly targeting common upstream molecular insults of *COL3A1* mutations. Recently, the FDA-approved chemical chaperone 4-phenylbutyric acid has shown promise in addressing endoplasmic reticulum (ER) stress and protein-folding defects in patient fibroblasts with glycine substitutions. As glycine substitutions are the most common pathogenetic variants [22], this highlights a potential therapeutic approach for addressing specific disease mechanisms associated with these prevalent alleles [23].

Although research has made significant strides in recent years, substantial progress remains ahead. From a biological perspective, there is still a limited understanding of the exact functional effects of the COLLIII defect within the extracellular matrix (ECM) and how alterations in the ECM influence the underlying mechanisms of disease. In this regard, we recently conducted a comprehensive transcriptome and miRNome survey on a large cohort of patient and control dermal fibroblasts, providing an in-depth view of the molecular alterations in vEDS. The transcriptome analysis identified 3189 differentially expressed genes (DEGs) involved in a complex pathological network, including disrupted ECM organization, altered proteostasis, ER stress-response failure, and defective autophagy, all of which are likely key drivers of the disease mechanisms. miRNome analysis suggested that a subset of DEGs could potentially be regulated by specific differentially expressed miRNAs (DE-miRNAs), including miR-15b-5p, miR-16-5p, miR-21-3p, miR-24b-3p, miR-29a-3p, miR-29b-3p, and others. Among these, the upregulation of the miR-29 family members, particularly miR-29a-3p and miR-29b-3p, appears to significantly disrupt ECM turnover and cell survival processes, including ER proteostasis and the autophagy-lysosome pathway, both of which are crucial for maintaining vascular integrity [24]. Elevated expression of miR-29a/b has already been shown to negatively affect key genes involved in vascular ECM structure and turnover, such as collagens (COLLs), elastin (ELN), and ECM-modifying enzymes, contributing to aortic damage and aneurysm development [25]. These miRNAs may thus represent promising molecular targets for vEDS, with a significant impact on vascular pathogenesis [22].

In the present study, we aimed to integrate the transcriptome findings by conducting a comprehensive proteomic analysis of vEDS patient fibroblasts. Additionally, we performed preliminary functional studies to explore the therapeutic potential of modulating miR-29b-3p activity. These investigations could lay the groundwork for developing alternative, tailored therapies aimed at alleviating the burden of vEDS and enhancing patient management strategies.

## 2. Materials and Methods

### 2.1. Patients

This study was approved by the local Institutional Review Board (ASST degli Spedali Civili, Brescia, Italy, registration number NP5328) and included 9 adult vEDS patients (P1-P9, 6 females and 3 males) and 9 unrelated age-matched healthy individuals (C1-C9, 7 females and 2 males) who were previously recruited for our transcriptome and miRNome study [24], providing written informed consent for participation and skin biopsy in accordance with the Italian bioethical standards. All patients underwent evaluation at the specialized outpatient clinic for Ehlers–Danlos syndromes and related connective tissue disorders at the University Hospital Spedali Civili of Brescia. Diagnoses were made following the 2017 EDS nosology [13], and patients were molecularly characterized in our laboratory for a pathogenic dominant negative *COL3A1* variant [14].

### 2.2. Cell Culture Conditions

Fibroblasts were cultured in our laboratory, following standard protocols, using skin biopsies collected from the same upper arm region of both vEDS patients and healthy donors [24]. The cells were maintained in vitro at 37 °C in a 5% CO_2_ atmosphere in Earle’s Modified Eagle Medium supplemented with 2 mM L-glutamine, 10% FBS, and 100 μg/mL penicillin–streptomycin (Thermofisher Scientific, Rodano, Italy). Fibroblasts were expanded to full confluency before being harvested using 0.25% trypsin/0.02% EDTA, ensuring a consistent passage number (between the 3rd and 5th) across samples.

Before conducting proteome analysis, we performed immunofluorescence analyses (IF) that verified the typical ECM component disorganization and integrin expression alteration in all analyzed patient fibroblast cell strains, consistent with our previous findings [26,27]. The disassembled ECM components included type I, III, and V fibrillar COLLs (COLLI, COLLIII, and COLLV); ELN, fibrillins (FBNs); fibronectin (FN); glycosaminoglycan chains (GAGs); and the core proteoglycans (PGs), i.e., perlecan, versican, and decorin. Furthermore, integrin expression analysis confirmed the absence of the α2β1 and α5β1 receptors and the presence of the αvβ3 integrin.

### 2.3. Label-Free Liquid Chromatography with Tandem Mass Spectrometry (LC-MS/MS)

For the proteomic experiment, 9 vEDS and 9 control cell strains were cultured in six-well plates with complete medium until reaching full confluency. Subsequently, cells were washed twice with PBS and lysed by incubating them for 20 min in RIPA buffer (20 mM Tris (pH 8.0), 150 mM NaCl, 0.1% SDS, 1% NP-40, and 0.5% sodium deoxycholate), supplemented with PhosSTOP and EDTA-free protease inhibitors. The lysates were centrifuged at 15,000 rpm for 20 min at 4 °C, and the resulting supernatant was collected. Protein concentration in the soluble fraction was determined using the colorimetric BCA protein assay kit (Thermofisher Scientific).

Protein extracts (30 µg) were precipitated with acetone, and then they were reduced and alkylated in a solution of 6 M guanidine-HCl, 5 mM TCEP, and 20 mM chloroacetamide. Proteins were digested into peptides by incubation with Lys-C for 3 h at 37 °C, followed by overnight digestion with the endopeptidase sequencing-grade trypsin (Promega Italia, Milano, Italy) at 37 °C. The resulting peptide mixtures were desalted and concentrated using the Stop-and-Go Extraction (STAGE) technique [28]. All experiments were conducted under label-free conditions. Peptide analysis was performed using a NanoLC 1200 system coupled to a quadrupole-based Q Exactive HF benchtop mass spectrometer via a nano-electrospray ionization source [29].

For the chromatographic separation, a binary buffer system was used, consisting of solution A (0.1% formic acid) and solution B (80% acetonitrile and 0.1% formic acid). The peptides were separated based on their hydrophobicity, using an analytical column (75 μm) packed in-house with C18-AQ 1.9 μm C18 resin. The separation process involved a gradient of 7–32% solvent B over 45 min, 32–45% B over 5 min, 45–95% B over 3 min, and finally 95–5% B over 5 min, with a flow rate of 300 nL/min.

MS data acquisition was performed in Data Independent Acquisition (DIA) mode, using 32 variable windows and covering a mass range of 300–1650 *m*/*z*. The resolution was set to 60,000 for MS1 and 30,000 for MS2. The AGC target was 3e6 for both MS1 and MS2, with maximum injection times of 60 ms for MS1 and 54 ms for MS2. The normalized collision energies (NCEs) were set to 25%, 27.5%, and 30%.

### 2.4. MS Data Processing and Analysis

Raw mass spectrometry data were processed using Spectronaut Software (2023 update). Protein assignment was performed by correlating spectra with the UniProt Homo sapiens database (UP000005640). Searches were conducted with tryptic specifications and default settings for mass tolerances for both MS and MS/MS spectra. Carbamidomethylation was set as a fixed modification, while oxidation and N-terminal acetylation were defined as variable modifications. The minimum and maximum peptide masses were set, respectively, to 7 and 470 Da, and the false discovery rate (FDR) for proteins and peptide-spectrum matches to 1%. Protein quantities were exported and additionally elaborated using the Perseus software (1.6.2.3). Specifically, the first level of data filtering was applied to exclude contaminant proteins/peptides, reverse entries, and the identification only by a modified peptide. The signal/noise values were normalized using log2 transformation, while the protein abundances were grouped according to experimental conditions. Missing values were replaced by random numbers drawn from a normal distribution. Differences between two groups were inspected using an unpaired Student’s *t*-test, and feature changes with q-value < 0.05 and log2 fold change ≥ ±1 were considered significant.

### 2.5. Bioinformatic Analyses

The biological interpretation of proteome changes was performed using Gene Ontology (GO) and Kyoto Encyclopedia of Genes and Genomes (KEGG) terms, using the Enrich database. Fisher’s exact test was used to calculate enrichments in categorical terms, with a Benjamini–Hochberg FDR < 0.05 set as the threshold. Clustering analysis was conducted with STRING functional protein association networks software (version 12.0) to evaluate significant protein–protein interaction networks related to the identified DEPs. Biological network construction was carried out using the ClueGO and CluePedia plugins of the Cytoscape platform (version 3.10.2), clustering dysregulated proteins based on their association with GO terms, WikiPathways, KEGG, and REACTOME pathway annotation terms. To predict target genes of miR-29b-3p, the PicTar, DIANA, TargetScan, and miRanda algorithms were used via the miRNAtap R package. Data visualization was performed in the R statistical environment (version 4.4.0), using the following packages: readxl, ggplot2, pheatmap, ropls, scales, dplyr, and gridExtra.

### 2.6. Cells Transfection

Dermal fibroblasts from 3 vEDS patients were seeded in complete culture medium at a density of 2.5 × 10^5^ cells/mL per well in a 6-well plate. The cells were transfected after 24 h using the HiPerFect transfection reagent, following the manufacturer’s instructions (Qiagen, Milano, Italy), with the following miRNAs at the final concentration of 150 pmol: anti-miRNA LNA inhibitor of hsa-miR-29b-3p (#YI04101843-DDA, inhibitor sequence: ACTGATTTCAAATGGTGCT), or negative control (NC) miRNA (#YI00199006-DDA, scrambled sequence: TAACACGTCTATACGCCCA). The culture medium was replaced 24 h after transfection, and cells were harvested at 48 and 72 h after transfection for RNA and protein purification. Transfection efficiency was assessed using the hsa-miR-1-3p miRCURY LNA miRNA Mimic (#YM00472818-ADA, mimic sequence: UGGAAUGUAAAGAAGUAUGUAU), as this miRNA is not expressed in dermal fibroblasts, and its inhibitory effect was verified by quantifying the expression levels of the *HDAC4* target gene.

### 2.7. mRNA and miRNA Expression Analyses

Total RNA, including miRNAs, was extracted from control and vEDS fibroblasts using the miRNeasy Mini Kit, according to the manufacturer’s instructions (Qiagen). To quantify the blood levels of hsa-miR-29b-3p, whole blood was collected in PAXgene Blood RNA Tubes (PreAnalytix, Qiagen), and RNA was isolated using the PAXgene Blood microRNA Kit, following the manufacturer’s instructions (Qiagen). RNA quality and concentration were assessed with the Agilent 2100 Bioanalyzer (Agilent Technology, Cernusco sul Naviglio, Italy) and the Qubit fluorometer (Thermofisher Scientific).

The relative expression levels of a subset of hsa-miR-29b-3p target genes were confirmed through qPCR, as previously reported [24]. Briefly, RNA samples were reverse transcribed with random primers, and qPCR was performed with SYBR Green dye and standard thermal cycling conditions on the QuantStudio 3 Real-Time PCR System (Thermofisher Scientific). *ATP5B*, *CYC1*, and *RPLP0*, were used for normalization. Primer sequences of target and housekeeping genes are reported in Supplementary Table S1.

To quantify the hsa-miR-29b-3p expression levels, 20 ng of total RNA was reverse-transcribed using the miRCURY LNA RT kit, following the manufacturer’s instructions (Qiagen). qPCR was carried out with the miRCURY LNA miRNA SYBR Green PCR kit and miRCURY LNA miRNA PCR assay specific for hsa-miR-29b-3p (assay ID: YP00204679). *SNORD48* (assay ID: YP00203903), *U6 snRNA* (assay ID: YP00203907), *SNORA66* (assay ID: YP00203905), *SNORD44* (assay ID: YP00203902), and *SNORD38B* (assay ID: YP00203901) reference miRNAs were amplified for normalization. Each sample was measured in technical triplicates. Relative mRNA and miRNA expression levels were normalized to the geometric mean of reference genes and analyzed using the 2^−(ΔΔCt)^ method. Statistical data were obtained with GraphPad Prism 8.0 by applying the unpaired Student’s *t*-test, with *p* < 0.05 considered statistically significant.

### 2.8. Protein Quantification by Immunoblotting

The expression levels of COLLI and COLLV(α1) were evaluated by Western blotting (WB) on pooled extracts from untransfected, miR-29b inhibitor-transfected, and NC-transfected fibroblasts from 3 vEDS patients, as well as untransfected cells from 3 age- and sex-matched healthy donors. Control and patient cells were lysed with RIPA buffer (50 mM Tris HCl pH 7.4, 150 mM NaCl, 0.25% Na-DOC, 1% NP-40, 1 mM EDTA, 0.02 UI/mL aprotinin, 5 μg/mL leupeptin, 1 μg/mL pepstatin, 10 mM NaF, 5 mM Na3VO4, and 1 mM PMSF) and centrifuged at 13,000 rpm at 4 °C for 10 min. The protein concentration of each pool was determined using the detergent compatible Bio-Rad Dc Protein Assay (Sigma Aldrich, Milano, Italy, #1001-491004), and each pool was loaded in triplicate.

To analyze COLLI, 35 μg of proteins was separated under reducing conditions by 8% SDS-PAGE electrophoresis. After nitrocellulose membranes transfer, the membranes were blocked overnight at 37 °C in 5% non-fat dry milk/TBS-0.1% (*v*/*v*) Tween 20 (TBS-T) and then immunoreacted for 3 h at room temperature (RT) with 1:500 goat anti-human COLLI antibody (Merck Life Science, Milano, Italy, #AB758) diluted in 5% non-fat dry milk/TBS-T. For COLLV(α1), 70 μg of proteins was resolved by 8% SDS-PAGE electrophoresis. The membranes were blocked for 3 h at RT in 5% non-fat dry milk/TBS-T and immunoreacted overnight at +4 °C with 1:1000 rabbit anti-human COL5A1 antibody (US Biological Life Science, Salem, MA, USA, #221804) diluted in 5% non-fat dry milk/TBS-T. After washing in TBS-T, membranes were incubated for 3 h at RT with HRP-conjugated anti-goat IgGs (Merck Life Science, #401515) and anti-rabbit IgGs (Merck Life Science, #A8275), respectively, diluted 1:1000 in 5% non-fat dry milk/TBS-T. Chemiluminescent signals were developed using the ECL method (Thermofisher Scientific, #34580). Band quantification was performed using the Image Pro Plus software version 6.2 (Media Cybernetics), and the integrated optic density values were normalized against the total amount of proteins transferred on the membrane in the same lane, stained by Sypro Ruby Protein Blot Stain (Thermofisher Scientific, #S-11791). Data analyses were performed with GraphPad Prism 8.0 software. Data are reported as means ± SEM. Differences were tested using Student’s *t*-test, and results with *p* < 0.05 were considered statistically significant.

### 2.9. Artificial Intelligence (AI)

In developing this work, the authors employed ChatGPT (Version 4, extension 3) by OpenAI to assist with grammar and syntax refinement. All content was subsequently reviewed and carefully edited by the authors, who accept full responsibility for the accuracy and integrity of the final publication.

## 3. Results

### 3.1. Exploring the Proteomic Landscape of vEDS Patient Fibroblasts and Its Integration with Transcriptomic Insights

Building on our previous transcriptomic study of vEDS fibroblasts [24], this research aimed to explore the proteomic landscape to identify altered protein networks and regulatory mechanisms potentially involved in the disease pathogenesis, while also integrating these findings with the transcriptomic data to provide a more comprehensive view of the molecular mechanisms driving vEDS.

**Figure 1:**
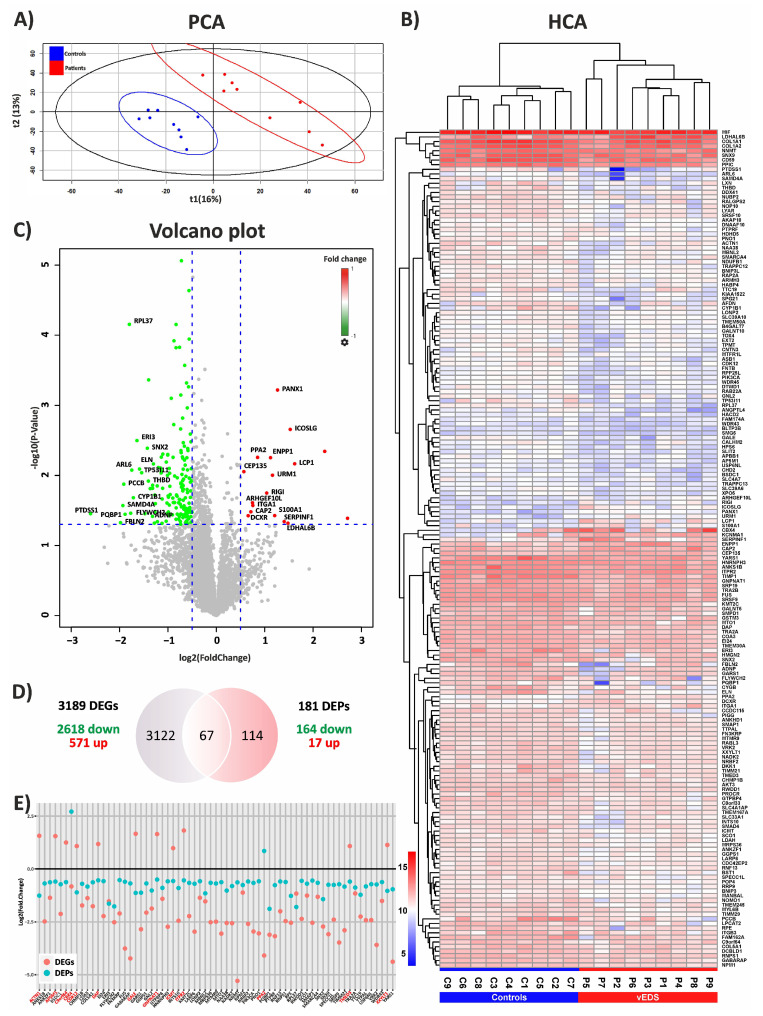
Differential protein expression analysis and integration with transcriptome data. (**A**) A 2D PCA plot showing the distribution of control (blue dots) and vEDS (red dots) samples in two distinct clusters. (**B**) Hierarchical clustering analysis (HCA) of the 181 DEPs identified in vEDS (P1–P9) vs. control (C1–C9) fibroblasts. Proteins with high expression values are shown in red, while those with low expression values are in blue. Each column of the heatmap represents a patient and control sample, while rows correspond to identified DEPs (Student’s *t*-test and FDR *p* < 0.05). (**C**) A Volcano plot displaying the distribution of the 164 downregulated (green) and the 17 upregulated (red) DEPs. The plots represent expression values as fold change (log2, *x*-axis) plotted against the -log10 FDR-adjusted *p*-value (*y*-axis). Protein symbols for a selection of DEPs are indicated. (**D**) A Venn diagram comparing the 3189 DEGs from the previous transcriptome study [13] with the 181 DEPs identified in the current proteomic study, which identified 67 shared genes/proteins. (**E**) A dispersion plot showing the differential expression of these 67 DEGs/DEPs, with red indicating opposite expression trend between the two omics approaches.

Using a label-free LC-MS/MS approach, we analyzed the intracellular proteome of 9 patient-derived fibroblast samples and 9 control samples, both subsets selected from the 18 vEDS and 36 control samples used in the original transcriptomic study. This proteomic analysis identified a total of 5485 proteins. After filtering for those present in at least 70% of samples per group, 5058 proteins remained. Differential expression analysis between vEDS and control fibroblasts revealed 181 DEPs, with 164 downregulated and 17 upregulated (Supplementary Table S2). Principal component analysis (PCA) distinctly differentiated vEDS patients from healthy individuals, as illustrated in Figure 1A. Consistent with the PCA results, the heatmap showed two distinct clusters clearly separating patients from healthy donors (Figure 1B). Proteins with high and low fold changes, along with significant *p*-values, are represented as red and green dots, respectively, in the Volcano plot (Figure 1C). PANTHER functional enrichment analysis of the 181 DEPs showed significant changes across various protein classes, with the most notable being metabolite interconversion enzymes (24%) and RNA metabolism proteins (16%). Other affected categories included protein binding-activity modulators (9%), protein-modifying enzymes (7%), scaffold/adaptor proteins (7%), transporters (7%), cytoskeletal proteins (5%), translational proteins (3%), ECM proteins (3%), and several others, each accounting for 1–2% (Supplementary Figure S1 and Supplementary Table S3).

To integrate the proteomics results with the transcriptomics data of GSE239914 [24], both a Venn analysis and a dispersion plot comparison were performed. When matching the 3189 DEGs from transcriptomics (2618 downregulated and 571 upregulated) to the 181 DEPs from proteomics, we identified 67 shared genes and proteins (Figure 1D). Dispersion plots further revealed that most (54) of these 67 overlapping molecules were consistently downregulated at both the transcriptional and translational levels, while 13 showed opposite expression changes (Figure 1E and Supplementary Table S4).

Overall, despite differences in sample sizes and sensitivities between the two omics approaches, this integrative analysis revealed a significant overlap in expression patterns, underscoring the biological relevance of both datasets and offering a clearer perspective on the underlying mechanisms driving vEDS pathogenesis by uncovering 114 DEPs not detected at the transcriptional level (Supplementary Table S4).

To elucidate the biological significance of the proteome changes, we performed a functional enrichment analysis on both the 54 overlapping genes/proteins and the 114 unique DEPs.

**Figure 2:**
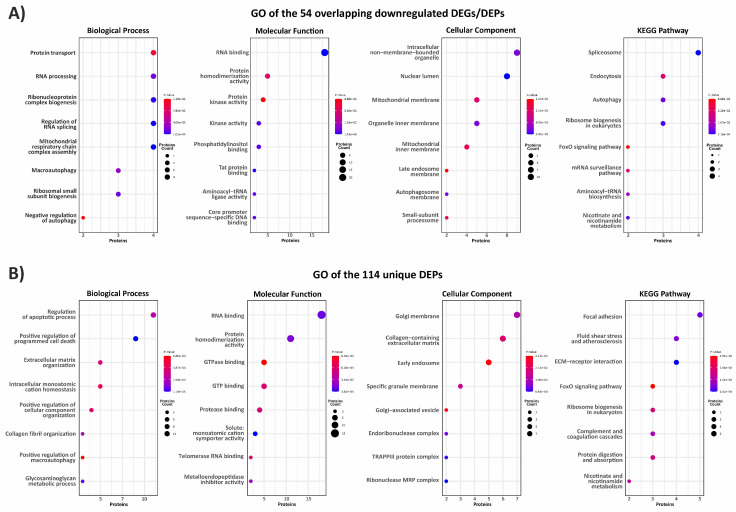
Functional enrichment analysis. (**A**) GO terms and KEGG pathways associated with the 54 overlapping downregulated genes/proteins and (**B**) those for the 114 DEPs identified exclusively in the vEDS proteome. The size of the dots reflects the number of proteins enriched in each annotation term, and the color represents statistical significance (−log10 *p*-value). The complete list of GO categories and KEGG pathways is provided in Supplementary Table S5.

As illustrated in Figure 2A, our GO analysis of the common downregulated DEPs/DEGs revealed enrichments in various biological processes and molecular functions mainly involved in protein transport/binding (e.g., NPM1, SNX2, SNX9, FUS, NADK2, GARS1, and SMARCA4); RNA processing/splicing/binding (e.g., MBNL2, GTPBP4, PNO1, KMT2C, YARS1, GNL2, MTO1, WDR43, GTPBP4, LARP6, ERI3, TRA2A, RNPS1, RPL37, HNRNPH3, PQBP1, and SRSF9); regulation of autophagy (e.g., CHMP1B, EI24, GABARAP, MTMR9, and PIK3CA); and mitochondrial functions, including the assembly of the respiratory chain complex (e.g., COA3, SCO1, TIMM21, and TTC19). The most enriched cellular components were intracellular non-membrane-bound organelles, nuclear lumen, and mitochondrial membrane. Spliceosome, endocytosis, autophagy, ribosome biogenesis, FoxO signaling pathway, mRNA surveillance pathway, aminoacyl-tRNA biosynthesis, and nicotinate and nicotinamide metabolism were the most enriched KEGG pathways.

The 114 proteins exclusively identified in the proteome were primarily associated with biological processes related to the regulation of the apoptosis and autophagy (BNIP3L, BNIP3, APBB1, CALHM2, CYP1B1, FAM162A, KCNMA1, SLIT2, SMPD1, ANGPTL4, DKK1, and WDR45), as well as ECM organization, collagen fibril organization, and GAG metabolic process (COL1A1, COL1A2, ELN, SERPINF1, FBLN2, CYP1B1, LCP1, B4GALT7, EXT2, GALNT5, and GALNT10). Among the molecular-function categories, RNA binding (e.g., SRP19, SLC4A1AP, POP4, SAMD4A, DDX41, CHD2, RPP25L, ANKHD1, SMG5, RRP9, TRA2B, HABP4, HMGN2, LYAR, SRSF10, and NOP10) was the most represented, with protein homodimerization activity (e.g., GSTM3, EXT2, SMAD4, BLTP3B, S100A1, RABL3, and ENPP1), GTP(ase) binding (e.g., RAP2A, ARL6, RABL3, RAB22A, AKT3, RIGI, XPO5, CDC42EP2, and HPS6), and metalloendopeptidase inhibitor activity (e.g., LXN and TIMP1) also being prominent. In terms of cellular compartments, the most enriched terms were those involved in vesicular trafficking from the ER to the Golgi, such as the TRAPPIII protein complex and the early endosome, followed by the collagen-containing ECM. According to the KEGG database, the most enriched pathways included focal adhesion and ECM–receptor interactions, fluid shear stress and atherosclerosis, FoxO signaling pathway, ribosome biogenesis, complement and coagulation cascades, protein digestion and absorption, and nicotinate and nicotinamide metabolism (Figure 2B).

To achieve a comprehensive understanding of the protein interaction network involving all 181 DEPs, including the 13 with expression patterns opposite to the transcriptomic analysis (Figure 1E), we employed an integrative approach using both STRING analysis and ClueGO enrichment.

**Figure 3:**
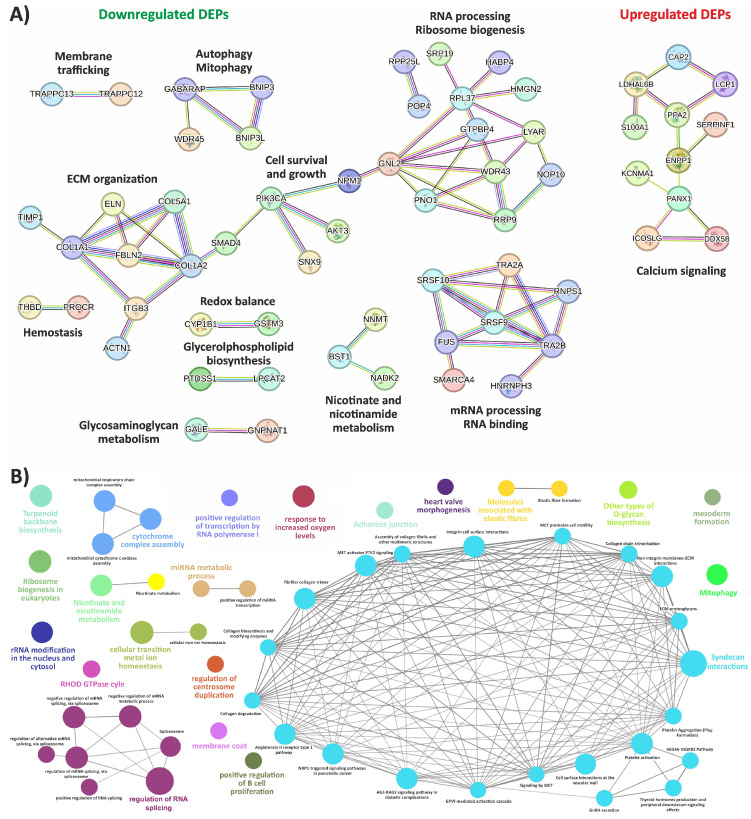
High-confidence protein–protein interactions and biological network enrichment of all DEPs. (**A**) STRING functional protein interaction networks of down- and upregulated proteins. Each node represents a protein, and each edge represents an interaction, including both physical and functional associations. Only interactions with a high confidence score (0.7) are shown. (**B**) Enriched biological networks, integrating GO terms with pathways from WikiPathways, KEGG, and REACTOME, using the ClueGO plugin in Cytoscape. Nodes represent enriched terms, connected by edges based on their κ score (≥0.7), reflecting term-term interactions and grouping into functional clusters based on shared genes. Node size indicates statistical significance, with only terms meeting a Benjamini–Hochberg-adjusted *p*-value threshold of ≤0.05 included.

The analysis of central nodes from biological networks, generated via the STRING database, using all the 164 downregulated DEPs, confirmed that key impaired biological processes in vEDS cells include RNA binding and processing, ribosome biogenesis, and ECM organization, all linked to mechanisms of cell survival and growth. Additionally, processes such as autophagy and mitophagy; membrane trafficking; cell redox balance; hemostasis; and the metabolism of glycerophospholipid, GAG, and nicotinate/nicotinamide were also identified within the biological networks. Regarding the 17 upregulated proteins, the only high-confidence network formed primarily comprised proteins associated with calcium signaling (Figure 3A).

To delve deeper into the biological terms and pathways associated with the changes in protein expression, we used ClueGO, a Cytoscape plugin that integrates GO and KEGG pathway enrichment to visualize non-redundant biological terms in a network format, providing a comprehensive overview of the most significant pathways and their interrelationships (Figure 3B and Supplementary Table S6). This analysis revealed that the most interconnected biological network was primarily linked to the maintenance of ECM homeostasis, the disruption of which may lead to connective tissues fragility, a hallmark of the disease. Within this network, molecular functions and pathways related to collagen biosynthesis, modification, fibril assembly, and degradation were indeed prominently represented. Other key terms in this network included ECM PGs and syndecan interactions, which mediate cell–matrix adhesion and modulate several signaling cascades involved in ECM organization. Integrin-mediated and non-integrin membrane–ECM interactions were also enriched, suggesting impairments in cell–ECM adhesion and associated signaling pathways. These include both direct and indirect regulators, such as GPVI- and AGE-RAGE-mediated pathways, along with signaling by MET, NRP1, and the angiotensin II receptor type 1 pathway. These signaling cascades collectively regulate numerous cellular responses to stress and injury, including mechanical stress, a critical factor considering vascular fragility in vEDS patients. Further expanding on this network, enriched groups were identified involving proteins implicated in platelet activation and aggregation, as well as in the VEGFA-VEGFR2 signaling pathway, which regulates endothelial cell proliferation, migration, survival, and angiogenesis. This suggests a potential dysregulation in vascular development and repair, reflecting possible connections to vascular function abnormalities seen in vEDS patients. In this context, an enriched cluster was also identified involving proteins implicated in elastic fiber formation that are essential for the flexibility and resilience of tissues, particularly within the vasculature.

Beyond ECM- and vascular-related processes, the analysis revealed several clusters related to cellular energy balance and metabolism of amino acids, vitamins, and cofactors. This encompassed pathways such as metal ion homeostasis, terpenoid backbone biosynthesis, nicotinate and nicotinamide metabolism, and mitochondrial functions crucial for respiratory chain assembly and cytochrome complex formation, all of which are essential for sustaining cellular energy homeostasis. Furthermore, processes linked to selective autophagy of mitochondria (e.g., mitophagy) and cell death were identified, underscoring a role for energy dysregulation and cellular stress in the pathogenesis of vEDS. Moreover, significant enrichment was observed in biological clusters associated with RNA polymerase I-mediated transcription, mRNA splicing, miRNA transcription, rRNA modification, and ribosome biogenesis, suggesting that vEDS fibroblasts undergo alterations in gene expression regulation at multiple levels, contributing to disease’s complexity. A final notable cluster involved the cellular response to increased oxygen levels, a critical adaptive mechanism in the face of oxidative stress. This response might be particularly relevant in vEDS, where oxidative stress may play a significant role in disease pathology.

### 3.2. Exploring the Role of miR-29b-3p in Disease Mechanisms and Its Therapeutic Potential

Our previous miRNomic analysis (GSE239908) revealed 137 DE-miRNAs in fibroblasts from vEDS patients compared to controls, with 66 upregulated and 71 downregulated. The Targetome analysis on a subset of DE-miRNAs, i.e., miR-15b-5p, miR-16-5p, miR-21-3p, miR-24-3p, miR-29a-3p, miR-29b-3p, miR-138-5p, miR-145-5p, and miR-195-5p, suggested that these DE-miRNAs, among others, may play a significant role in regulating differential gene expression in the transcriptome. Specifically, upregulated miRNAs were associated with the downregulation of several target DEGs, likely due to mRNA degradation, whereas downregulated miRNAs correlated with the upregulation of target DEGs, possibly by reducing mRNA degradation. Among these DE-miRNAs, the elevated expression of miR-29a-3p and miR-29b-3p correlated with decreased levels of a group of putative target DEGs principally involved in ECM organization, ER homeostasis, and autophagy [24].

**Figure 4:**
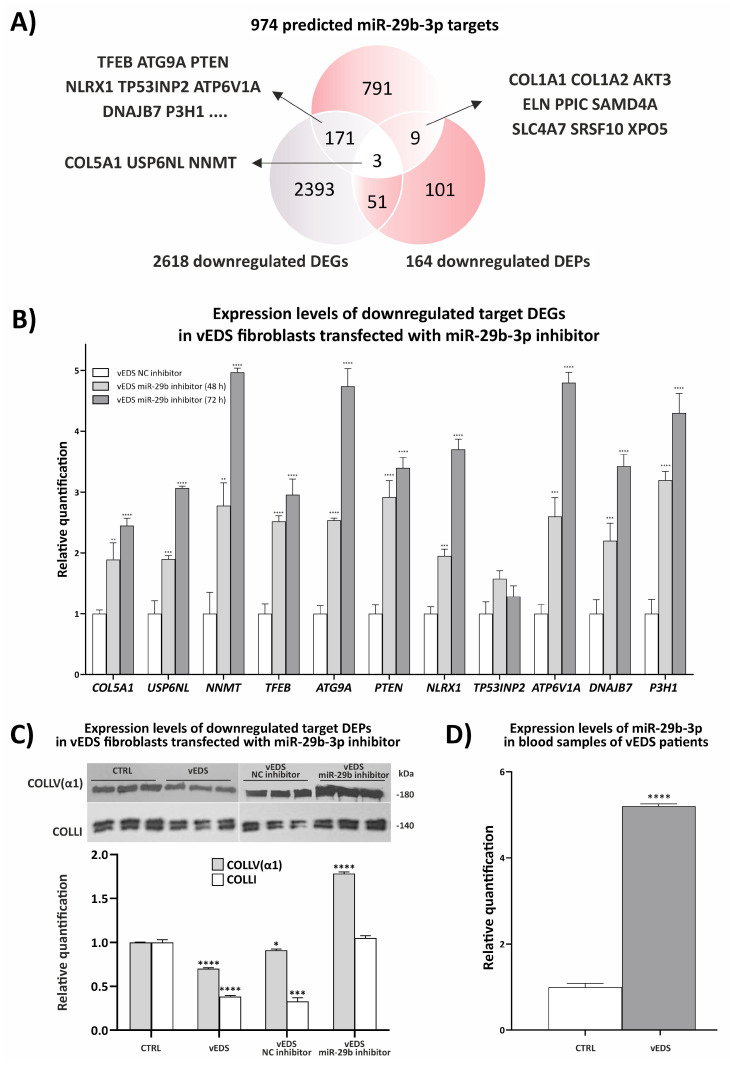
Deciphering the impact of miR-29b-3p on vEDS pathobiology. (**A**) A bioinformatics survey was conducted to predict the target genes of miR-29b-3p using the PicTar, DIANA, TargetScan, miRanda, and MiRDB algorithms. This analysis identified 974 non-redundant putative target genes for miR-29b-3p. A Venn diagram summarizes the integration of these 974 miR-29b-3p target genes with the 2618 downregulated DEGs and the 164 downregulated DEPs. (**B**) Expression levels of downregulated target DEGs were measured in vEDS fibroblasts transfected with a miR-29b-3p inhibitor. Patient cells were transfected with 150 pmol of a specific miR-29b-3p inhibitor or corresponding negative control (NC) for 48 and 72 h. The relative mRNA expression levels of miR-29b-3p target DEGs were quantified using the 2^−(ΔΔCt)^ method, with normalization to the geometric mean of the reference genes *ATP5B*, *CYC1*, *RPLP0*, and *YWHA.* Bars represent the mean expression ratios from pooled RNA samples of 3 vEDS and 3 control cells transfected with either the specific miR-29b-3p inhibitor or the corresponding NC. All qPCR analyses were performed in quadruplicate across two independent experiments. Statistical significance between groups was assessed using an unpaired Student’s *t*-test, and data are presented as mean ± SEM (** *p* < 0.01, *** *p* < 0.001, and **** *p* < 0.0001). (**C**) Representative WB images and quantitative protein analyses of COLLV(α1) and COLLI in untransfected control (n = 3) and vEDS fibroblast (n = 3), as well as in vEDS cells (n = 3) transfected with 150 pmol of miR-29b inhibitor or NC for 72 h. Graphical results are presented as mean ± SEM of technical triplicates. Statistical significance compared to untransfected control cells was assessed using Student’s *t*-test, (* *p* < 0.05, *** *p* < 0.001, and **** *p* < 0.0001). (**D**) Expression levels of miR-29b-3p in blood samples from vEDS patients (n = 12) and healthy individuals (n = 24) were quantified via qPCR, using the 2^−(ΔΔCt)^ method, with normalization to the endogenous controls *SNORD48*, *U6 snRNA*, *SNORA66*, *SNORD44,* and *SNORD38B.* Bars represent the mean expression ratios from pooled RNA of 12 vEDS and 24 control blood samples. All qPCR analyses were performed in quadruplicate across two independent experiments. Statistical significance between groups was assessed using an unpaired Student’s *t*-test, and data are presented as mean ± SEM (**** *p* < 0.0001).

Given the multifaceted roles of miRNAs in regulating gene expression, including their ability to inhibit protein translation without necessarily altering mRNA abundance [30], we aimed to investigate how specific miRNAs, particularly miR-29b-3p, influence the proteomic landscape of vEDS fibroblasts. By exploring the relationship not only between miRNA expression and target DEGs levels but also between miRNA expression and target protein levels, we sought to better elucidate the mechanisms by which miR-29b-3p may impact cellular processes relevant to ECM homeostasis and cell survival and stress responses. This proof-of-concept investigation, centered on miR-29b-3p but potentially extendable to other DE-miRNAs, such as miR-29a-3p, aimed to demonstrate the therapeutic potential of inhibiting upregulated miRNAs to modulate gene expression and restore impaired cellular functions contributing to the disease pathogenesis. To achieve this, we first queried various up-to-date miRNA-target databases, identifying a total of 974 potential target genes for miR-29b-3p (Supplementary Table S7). Next, as miRNAs primarily exert post-transcriptional repression, we performed a Venn analysis comparing these 974 predicted targets of miR-29b-3p with both the previously identified 2618 downregulated DEGs and the 164 downregulated DEPs from the present study. As shown in Figure 4A, this analysis revealed 3 mir-29b-3p putative target genes that showed reduced expression at both the RNA and protein levels, 9 that were downregulated only at the protein level, and 171 that exhibited reduced mRNA levels only. The three genes downregulated at both levels were *COL5A1*, *NNMT*, and *USP6NL*, which encode, respectively, the α1 chain of type V collagen essential for the structure and integrity of the ECM; nicotinamide N-methyltransferase involved in the metabolism of nicotinamide and various cellular processes, such as proliferation and differentiation; and ubiquitin-specific protease 6 N-terminal-like, which regulates autophagy, cell cycle progression, and apoptosis.

Among the nine downregulated target DEPs, for which miR-29b-3p likely inhibits translation without altering mRNA levels, there were key ECM components, such as COL1A1, COL1A2, and ELN. Other downregulated targets included PPIC (involved in protein folding), AKT3 (a serine/threonine protein kinase), SAMD4A (involved in RNA metabolism), SLC4A7 (a sodium bicarbonate transporter), SRSF10 (a splicing factor), and XPO5 (responsible for pre-miRNA nuclear export). Finally, among the 171 downregulated target DEGs, several were involved in autophagosome formation, the mTOR-autophagy–lysosomal signaling pathway, collagen biosynthesis, protein folding, and ER quality control. Notable examples, among others, include *TFEB* (a master regulator of autophagy), *ATG9A* (essential for autophagosome formation), *PTEN* (a key regulator of cell growth), *NLRX1* (an autophagy and inflammation modulator), *TP53INP2* (involved in stress responses), *ATP6V1A1* (important for pH regulation), *DNAJB7* (a chaperone for protein folding), and *P3H1* (involved in collagen biosynthesis). The absence of corresponding protein downregulation for these transcripts may result from the lower sensitivity of the proteomic analysis, which also involved fewer samples than the transcriptomic study. Additionally, compensatory mechanisms, such as increased translation efficiency, protein stability, or delayed translation responses, could also maintain protein levels despite reduced mRNA levels.

In view of these findings, we aimed to determine whether inhibiting miR-29b-3p in vEDS cells could influence the transcriptional levels of the downregulated putative target DEGs by transfecting patient fibroblasts with a specific miR-29b-3p inhibitor and quantifying the relative mRNA levels using qPCR. We first optimized the experimental conditions by transfecting vEDS cells with a control mimic (hsa-miR-1-3p) at concentrations ranging from 15 to 150 pmol. The qPCR results confirmed a dose-dependent decrease in the mRNA levels of its target gene, *HDAC4*, with the most significant reduction observed at the 150 pmol concentration (Supplementary Figure S2). Consequently, vEDS fibroblasts were then transfected with an inhibitor specifically targeting miRNA-29b-3p for 48 and 72 h at this concentration, alongside negative control (NC, scrambled miRNA) experiments. The qPCR confirmed a significant reduction in miRNA-29b-3p levels when treated with the inhibitor compared to NC (Supplementary Figure S3), demonstrating that the inhibitor successfully reduced miR-29b-3p activity, thus setting the stage for assessing its downstream effects on target gene expression. Indeed, we then investigated the expression levels of the three genes downregulated at both the RNA and protein levels (*COL5A1*, *USP6NL*, and *NNMT*), as well as the abovementioned target DEGs downregulated only at mRNA level and principally involved in ECM organization, autophagy, and ER homeostasis (*TFEB*, *ATG9A*, *PTEN*, *NRLX1*, *TP53INP2*, *ATP6V1A*, *DNAJB7*, and *P3H1*). As illustrated in Figure 4B, the inhibition of miR-29b-3p led to a significant increase in the expression levels of all DEGs, except for *TP53INP2*, with a more marked effect generally observed at 72 h. These findings demonstrate that miR-29b-3p effectively targets these specific mRNAs, inducing their degradation, and underscore its potential as a therapeutic target for modulating gene expression and restoring impaired cellular functions.

Finally, we validated, through WB, the protein levels of three representative miR-29b-3p targets, namely *COL5A1*, *COL1A1*, and *COL1A2*, in untransfected control and vEDS fibroblasts, as well as in patient cells transfected with 150 pmol of either miR-29b-3p inhibitor or NC inhibitor for 72 h. To accomplish this, we selected an antibody specifically recognizing the α1 chain of collagen type V [COLLV(α1)], which was found to be downregulated at both the RNA and protein level, and an antibody recognizing both the α1 and α2 chain of collagen type I (COLLI), which were found to be downregulated at the protein level only (Figure 4A). As shown in Figure 4C, WB validation confirmed that the levels of both COLLV(α1) and COLLI were significantly lower in patients’ fibroblasts compared to control cells.

Most notably, in contrast to cells transfected with the NC inhibitor, vEDS fibroblasts transfected with the miR-29b-3p inhibitor showed increased levels of both ECM components. Specifically, following miR-29b-3p inhibition, COLLI levels were comparable to those observed in untransfected control fibroblasts, while COLLV(α1) levels were even higher than those in control cells. These results provide the first evidence that miR-29b-3p regulates the protein levels of critical structural components essential for maintaining ECM integrity in vEDS patient fibroblasts via mechanisms of mRNA degradation and/or translational inhibition.

Building on these promising in vitro findings, we next sought to verify the expression levels of miR-29b-3p in blood samples from a small cohort of vEDS patients (n = 12), all of whom were molecularly characterized in our lab for a pathogenic dominant negative *COL3A1* variant [14]. As shown in Figure 4D, qPCR analysis revealed a significant upregulation of miR-29b-3p in the blood of vEDS individuals compared to healthy donors. Overall, these findings underscore the critical role of miR-29b-3p in the pathophysiology of vEDS, particularly in regulating ECM integrity and cell survival processes. In a translational context, our findings provide compelling proof of concept for further functional investigations aimed at assessing whether targeting miR-29b-3p in vEDS patients could be a viable therapeutic strategy for improving ECM homeostasis and critical intracellular functions, both of which are essential for maintaining connective tissue integrity, especially in the vasculature.

## 4. Discussion

Current treatments for vEDS are primarily symptomatic, reflecting our incomplete understanding of the underlying disease mechanisms. Detailed studies at the molecular and cellular levels are essential to identify biological targets and causative mechanisms driving the disease. Investigating how *COL3A1* mutations affect the structure and function of the ECM, as well as critical biological processes to maintain intracellular homeostasis, is pivotal for developing effective therapies and improving patient management.

In this study, we adopted an integrative approach by constructing a comprehensive proteomic map of patient dermal fibroblasts and integrating it with previously collected transcriptome and miRNome profiles. This multi-level perspective of the molecular landscape aimed to reveal a potential disease signature with translational relevance. By combining these datasets with proof-of-concept functional data, our goal was to gain deeper insight into deregulated pathomolecular mechanisms and explore the impact of the miR-29b-3p in vEDS pathogenesis. While we recognize that dermal fibroblasts may not be the optimal cell type to fully capture the in vivo alterations associated with the disease, and that endothelial cells or smooth muscle cells, for instance, would likely provide a better representation of the alterations in the vasculature, previous studies have successfully used this in vitro model to investigate cellular dysfunction and molecular pathways in various connective tissue disorders, including vEDS [23,24,26,27]. Indeed, integrated multi-omics strategies, whether in vitro or in vivo, can bolster translational research efforts by providing biological insights into the functional impacts of potential disease-associated biomolecules, as demonstrated in the context of other vasculopathies [31,32,33,34]. Additionally, the exploration of epigenetic alterations, including miRNA dysregulation, offers valuable clues for developing therapeutic strategies aimed at mitigating vascular diseases [31,35]. Thorough investigation by multi-omics and epigenetic analyses is crucial for unraveling the intricate regulatory networks underlying vEDS development, aiding in the identification of biomarkers for disease progression. Despite advances in understanding other vasculopathies [33], a notable gap remains in omics and epigenetic studies specifically focused on vEDS. This gap limits our biological understanding of disease processes and hinders the discovery of novel therapeutic targets. Addressing this gap and identifying key biomarkers and therapeutic targets would significantly enhance efforts to manage vEDS in clinical practice [22].

Consistent with our previous transcriptome results [24,26], the present proteome investigation reinforces our proposed disease model regarding the impact of dominant negative *COL3A1* variants on vEDS pathobiology [24]. Our current findings highlight that ECM structure and stability, autophagy/mitophagy, mitochondrial function, cellular redox balance, and apoptotic processes are key deregulated biological processes driving the underlying molecular pathophysiology. This is supported by enrichment analysis results indicating decreased expression of numerous proteins involved in these biological networks. Reduced protein levels of fundamental connective tissue components, prominently expressed in skin and blood vessels, such as COLLs (COL1A1, COL1A2, and COL5A1); elastic fiber components (ELN and FBLN2); ECM interactors and regulators, like integrins (ITGA1, ITGB3) and matrix metalloprotease inhibitors (TIMP1); and intracellular signal transducers (e.g., SMAD4, PIK3CA, and AKT3), underscore the substantial impact of altered assembly or availability of key ECM molecules and consequent ECM homeostasis perturbation on the development of connective tissue abnormalities, particularly within the vascular system. These deficiencies in critical ECM proteins may contribute to major vascular complications observed in affected individuals, including arterial rupture, thrombosis, dissection, or aneurysm, and abnormalities in elastin structure and function [8,14,18]. Additionally, key autophagy-related proteins (BNIP3, BNIP3L, GABARAP, NRBF2, and PIK3CA), along with proteins involved in mitochondrial function and cellular redox balance (NADK2, BST1, NNMT, and ENPP1), and apoptotic regulators (APBB1, BNIP3, CALHM2, CYP1B1, FAM162A, and KCNMA1), further illustrate the complexities of these dysfunctions. Indeed, pathological turnover of the ECM, coupled with altered autophagy, mitochondrial dysfunction, and activation of cell death programs, has been identified as a significant driver in various cardiovascular conditions [36], including multiple thoracic aortic aneurysm diseases, such as Marfan (MFS) and Loeys–Dietz syndromes (LDS) [32,37]. Notably, integrative analyses combining transcriptomic and proteomic approaches on aortic samples from MFS patients and fibulin-4 mutant mice revealed mitochondrial dysfunction and impaired oxidative phosphorylation as significantly affected biological functions, including downregulation of genes and proteins associated with mitochondrial respiration and metabolism [32,37]. While the precise contribution of mitochondria dysfunction to aortic aneurysm formation and vasculopathies is still being clarified, there is supporting evidence. For instance, our previous research on dermal fibroblasts from patients with arterial tortuosity syndrome demonstrated that the loss of function of the GLUT10 glucose transporter leads to reduced recycling of dehydroascorbic acid into the endomembrane systems, increased oxidative damage, and cellular energy imbalance [38,39]. Furthermore, fibroblasts from MFS and LDS patients carrying mutations in the *FBN1*, *TGFBR2*, and *SMAD3* genes show decreased oxygen consumption rates. These findings reinforce the idea that disrupted mitochondrial biogenesis and function could contribute to aneurysmal diseases [33,37]. Additionally, transcriptomic and metabolomic analyses of aortic samples from a murine model of MFS identified mitochondrial dysfunction as a significant contributor to the pathogenesis of aortic disease [40]. In accordance with this evidence, our proteomic results further support the view that perturbed mitochondrial metabolism might play a role in the pathogenesis of vascular connective tissue disorders. Moreover, impaired proteostasis; disrupted ER homeostasis; and dysregulated adaptive cellular mechanisms, such as the unfolded protein response [24], may contribute to the fragility and instability of blood vessels in vEDS, resulting in complications like arterial ruptures and other vascular abnormalities. Understanding how vEDS fibroblasts respond to intracellular alterations leading to ER stress and other downstream consequences, such as disturbed autophagic pathways, oxidative imbalance, and increased apoptosis, may provide important insights into potential druggable targets for enhancing cellular resilience and improving vascular health in vEDS patients. A recent study demonstrated the induction of selective autophagy of the ER (ER-phagy) in fibroblasts from kyphoscoliotic EDS patients as a cellular response to misfolded collagen accumulation, reinforcing the involvement of autophagy in EDS [41].

An essential question to consider is how a deficiency in a protein crucial for the structural integrity of the vascular ECM can result in changes in mitochondrial function and metabolism. The potential interaction among COLLIII deficiency, along with the reduced bioavailability of other ECM structural constituents (i.e., other fibrillar COLLs, FN, and ELN) [24,26], and mitochondrial dysfunction can be explained by the disturbance of ECM homeostasis. These alterations in ECM architecture and remodeling may induce changes in mechanotransduction, subsequently affecting the functional connections between the ECM and the organization of the cytoskeleton [42]. These altered ECM-to-cell signaling may be influenced by changes in cellular dynamics, including alterations in the adhesion properties of integrin receptors and cell adhesion complexes, as already described in vEDS patient fibroblasts [27]. Given that mitochondria depend on the cytoskeleton for their mobility and proper activities [42], disruptions in both ECM structural integrity and cytoskeletal organization could affect mitochondrial distribution, structure, and function [37]. Further studies should therefore focus on metabolomic analysis in vEDS patients to further explore and validate these initial findings, shedding light on diverse pathobiological aspects of the disease.

Our integrative multi-omics investigation revealed a complex interaction network, identifying several compromised molecular mechanisms and emphasizing the critical roles of miRNAs, particularly miR-29b-3p, in regulating key extracellular and intracellular biological processes, including ECM homeostasis, autophagy, and the ER stress response, all of which are likely implicated in the pathogenesis of vEDS [24,27,43]. While the exact functional role of miR-29b in the context of vEDS remains largely unexplored, our findings suggest it may be a critical player in disease progression and could inform targeted therapy development. Consistent with evidence from other vascular disorders [25,31,44,45], miR-29b-3p likely holds significant pathological relevance in vEDS as well. Indeed, our in vitro functional data and preliminary in vivo results suggest that miR-29b-3p acts as a negative regulator of the ECM homeostasis and related cellular pathways. The inhibition of miR-29b-3p in patient fibroblasts correlated with the upregulation of several target DEGs, including genes encoding ECM structural components and those involved in adaptive cellular responses, alongside increased protein levels of COLLV(α1) and COLLI. These in vitro findings, together with our pilot in vivo results showing higher miR-29b-3p levels in patients compared to healthy donors, position this miRNA as a promising target for enhancing ECM integrity and intracellular homeostasis. Inhibiting miR-29b-3p could have therapeutic potential by addressing these alterations, especially given its well-documented pathological role in vascular ECM remodeling across various conditions. Indeed, previous studies on human thoracic aortic aneurysm samples have shown a strong association between increased miR-29-3p expression and the downregulation of several ECM components, including COLLs and ELN. This downregulation contributes to the weakening of the aortic structure, ultimately facilitating aneurysm development [46]. In murine models of abdominal aortic aneurysms, miR-29b-3p upregulation accelerated aneurysm expansion and increased rupture rates, accompanied by decreased expression of key ECM targets (e.g., *Col1a1*, *Col3a1*, *Col5a1*, and *Eln*), while inhibition of miR-29b-3p reduced aortic aneurysm progression [47]. Similarly, in MFS mice models, miR-29b-3p upregulation in the ascending aorta led to ECM degradation through increased matrix metalloproteinase activity [48]. These findings highlight the importance of miR-29b-3p in pathological vascular ECM remodeling, further suggesting it may be a key molecular driver of vEDS pathogenesis.

Importantly, emerging therapeutic strategies for miRNA inhibition are showing great promise in several preclinical studies [49]. Specifically, miRNAs can be effectively targeted in vivo using advanced delivery systems such as lipid nanoparticles (LNAs), which ensure stability in the bloodstream, demonstrating efficacy in reducing miRNA levels and modulating disease pathways in several conditions, including cardiovascular disorders [50,51,52]. Additionally, further strategies, including peptide-conjugated phosphorodiamidate morpholino oligomers (PMOs), antisense oligonucleotides (ASOs), and locked nucleic acids (LNAs), are being explored for therapeutic applications in various diseases [49,52]. By addressing miR-29b’s role in ECM dysregulation, these innovative strategies offer promising therapeutic avenues that could improve vascular integrity and mitigate disease progression in vEDS.

Taken together, while our in vitro and in vivo findings are promising, further validation and research is mandatory to confirm clinical relevance and therapeutic potential. Moreover, a deeper understanding of the deregulated pathways will likely reveal further potential therapeutic targets and aid in the development of more effective treatment strategies. In conclusion, our research may serve as a foundation for future studies focused on creating targeted treatments to meet the urgent need for improved interventions in this life-threatening disorder.

## Data Availability

Most data generated or analyzed during this study are included in this published article and its Supplementary Materials. Additional data and materials are available from the corresponding authors upon reasonable request, subject to compliance with our obligations under human research ethics.

## Supplementary Materials

The following supporting information can be downloaded at https://www.mdpi.com/article/10.3390/biomedicines12122749/s1. Figure S1: Altered protein classes in vEDS fibroblasts according to the PANTHER protein classification database. Figure S2: Optimization of transfection conditions in vEDS patient and control fibroblasts. Figure S3: Validation of miR-29b-3p inhibition. Table S1: Primer sequences of miR-29b-3p target genes. Table S2: Proteomics results of vEDS fibroblasts. Table S3: GO classification of the DEPs according to PANTHER database. Table S4: Integrative analysis of transcriptomic and proteomic results. Table S5: GO classification according to the Enrich database. Table S6: Network enrichment according to ClueGO. Table S7: Target analyses of miR-29b-3p.
