# Supplementary material for: Integrative Multi-Omics Approach in Vascular Ehlers–Danlos Syndrome: Further Insights into the Disease Mechanisms by Proteomic Analysis of Patient Dermal Fibroblasts
Source: Biomedicines. 2024 Nov 30;12(12):2749. doi: 10.3390/biomedicines12122749 (PMC11727028; doi:10.3390/biomedicines12122749)
Supplement: Supplementary file 1 [file biomedicines-12-02749-s001.zip › Supplementary Figures.pdf]

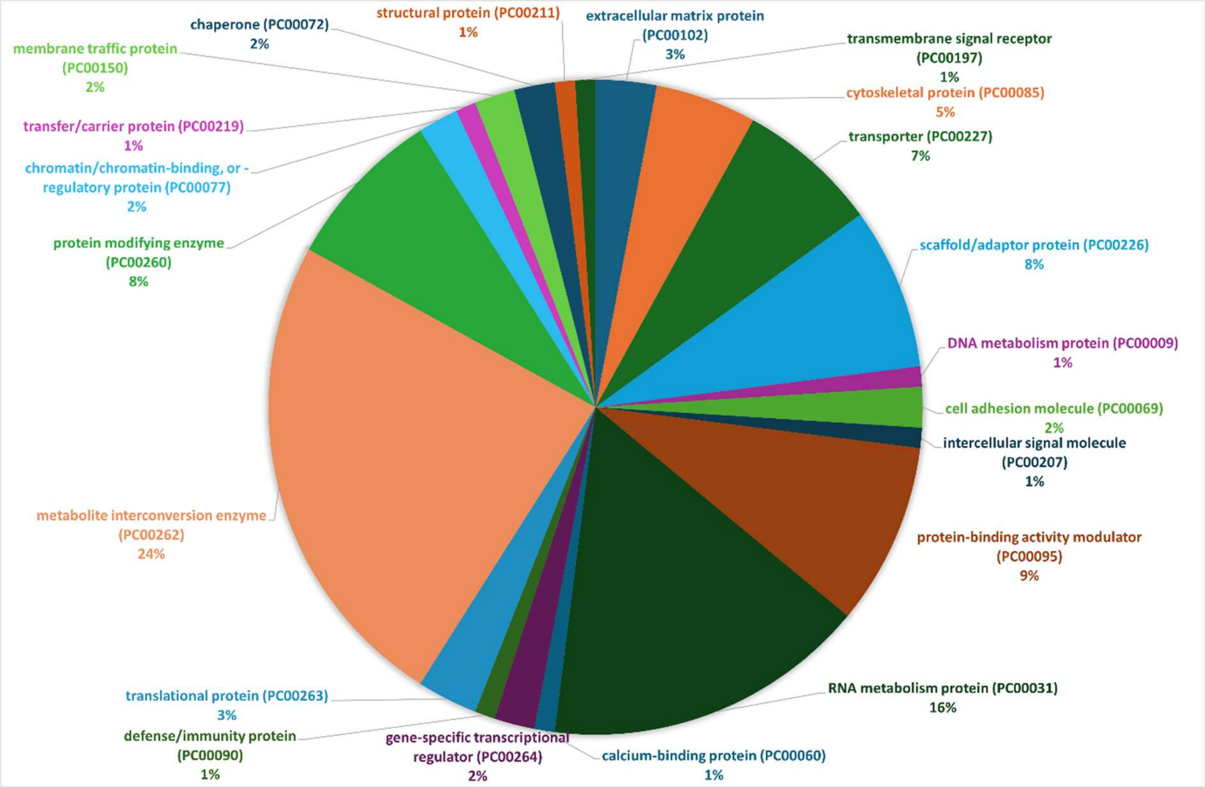

Supplementary Figure S1. Altered protein classes in vEDS cells according to the PANTHER protein classification database

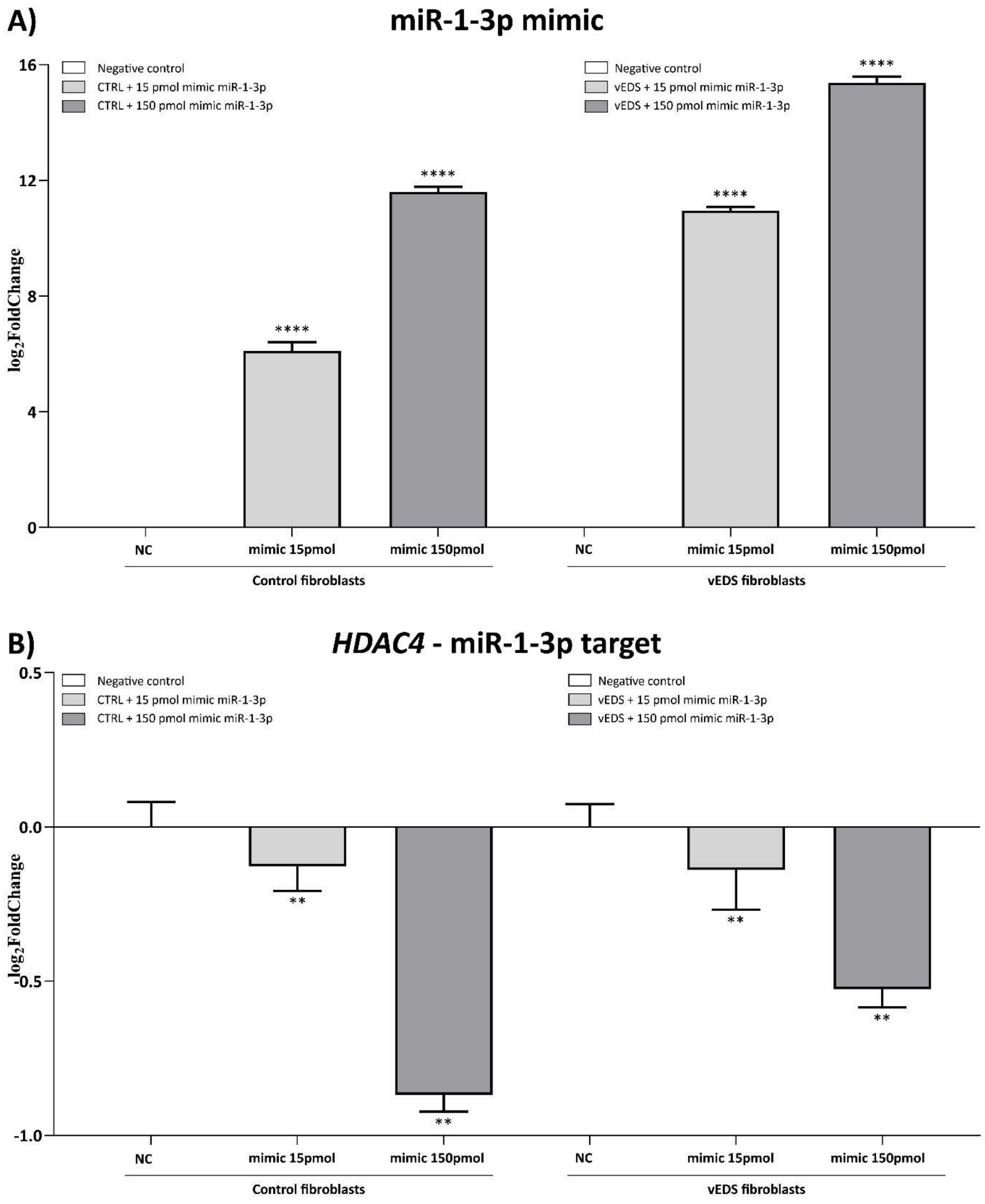

Supplementary Figure S2. Optimization of transfection conditions was performed in patient and control fibroblasts, which were transfected for 48 and 72 hours with 15 and 150 pmol of the hsa-miR-1-3p and the corresponding control microRNA inhibitor (negative control). qPCR confirmed a dose-dependent increase in miRNA expression in both vEDS and control cells (**A**). The efficiency of miR-1-3p overexpression was evaluated by analyzing the relative mRNA levels of its target gene, *HDAC4*. qPCR showed a dose-dependent decrease in *HDAC4* expression compared to the control (**B**). Expression levels of miR-1-3p and *HDAC4* were quantified using the $2^{(-\Delta\Delta Ct)}$ method. For miR-1-3p, the reference miRNAs *SNORD48*, *U6 snRNA*, *SNORA66*, *SNORD44*, and *SNORD38B* were used as endogenous controls for template normalization. For *HDAC4*, the geometric mean of the reference genes *ATP5B*, *CYC1*, *RPLP0*, and *YWHAZ* was used for cDNA input normalization. Statistical significance between groups were determined using an unpaired Student's t-test and data are expressed as mean ± SEM (** p < 0.01, ****p < 0.0001).

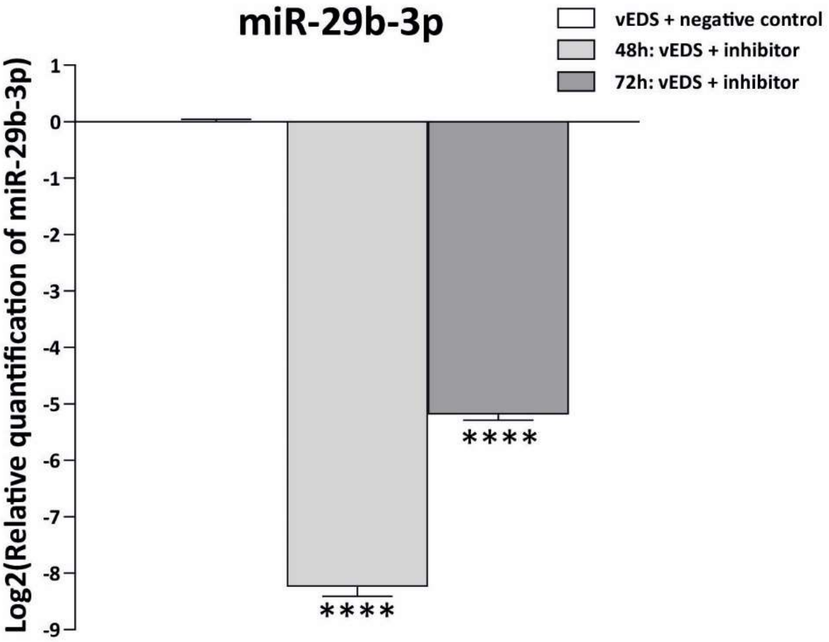

Supplementary Figure S3. Validation of the inhibition of miR-29b-3p: Patient cells were transfected with 150 pmol of a specific miR-29b-3p inhibitor or corresponding negative controls (scramble) for 48 and 72 hours. The efficiency of miR-29b-3p inhibition was assessed by measuring a time-dependent decrease in expression. miR-29b-3p expression levels were quantified using qPCR with the $2^{\wedge-(\Delta\Delta Ct)}$ method. *SNORD48*, *U6 snRNA*, *SNORA66*, *SNORD44*, and *SNORD38B* were used as endogenous controls for template normalization. The bars represent the mean expression ratios from pooled RNA samples of three vEDS cells transfected with either the specific miR-29b-3p inhibitor or the corresponding negative controls. Statistical significance was determined using the Student's t-test (**** $p < 0.0001$).
