# Supplementary material for: Integrative Multi-Omics Approach in Vascular Ehlers–Danlos Syndrome: Further Insights into the Disease Mechanisms by Proteomic Analysis of Patient Dermal Fibroblasts
Source: Biomedicines. 2024 Nov 30;12(12):2749. doi: 10.3390/biomedicines12122749 (PMC11727028; doi:10.3390/biomedicines12122749)
Supplement: Supplementary file 1 [file biomedicines-12-02749-s001.zip › Table S1.pdf]

Supplementary Table S1: Primers sequences of miR-29b-3p target genes						
**Genes**	**RefSeq**	**Product size**	**Primer forward**	**Tm**	**Primer reverse**	**Tm**
COL5A1	NM_000093.4	116	GATGACCTCACCTATGGCGA	58.96	GGAGCTGGATTGGAGGAGTT	59.08
USP6NL	NM_014688.5	135	TGACAGAACTAAAGCGGGCA	61.9	GCTCCTCTGTCCGTTGCTAA	60.5
NNMT	NM_006169.3	119	CCCCAGTGGTGACCTATGTG	61.2	GCTGGCTCTGAGTCACATCA	60.1
TFEB	NM_001167827.3	105	GGAGGTGTTGAAGGTGCAGT	60.16	TTCCCATAGGTCTCGGACAG	60.07
ATG9A	NM_001077198.3	158	ATCCCCAGTGGCTATCTGCT	61.1	CTGCTCCTTGAGGAAGCCTA	59.7
PTEN	NM_000314.8	170	CGGAACTTGCAATCCTCAGT	60.25	GCATCTTGTTCTGTTTGTGGAA	60.15
NLRX1	NM_001282144.2	141	GCCCCCTAGGGCCTTTATAC	60.9	CTGAACCACTCAGCCAGGTT	60.3
TP53INP2	NM_021202.3	111	ACCTGCCGGACAGCTACG	62.4	GAGGGGTAACAAACCAGCTC	68.6
ATP6V0A1	NM_001378530.1	143	TGGAGGATCCTGTGACTGTG	59.66	GCCCTGTAATCCCCAGCTAC	60.85
DNAJB7	NM_145174.1	84	CGTGGACAATGATGAGGGAGG	60.47	CCACCCTCTTTGACTCCTGC	60.32
P3H1	NM_022356.4	189	GCAGGTCGCCTACTTCAAGAT	60.78	CCCAGTCGAAATTCTTGCAT	60.08
ATP5B	NM_001686.3	127	CCAATTCTAAATGCCCTGGA	59.89	GGCCTCTAACCAAGCCTTCT	59.85
CYC1	NM_001916.3	147	CTACGGACACCTCAGGCAGT	60.32	CAGGTCACTGGCACTCACAG	60.52
RPLP0	NM_001002.3	135	GCAATGTTGCCAGTGTCTGT	59.76	CCTTTTCAGCAAGTGGGAAG	59.85
HDAC4	NM_001378414.1	103	AGGCTCAGACTTGCGAGAAC	59.80	ATGGGCTCCTCATCTGGTCT	61.00
