# Supplementary material for: Integrative Multi-Omics Approach in Vascular Ehlers–Danlos Syndrome: Further Insights into the Disease Mechanisms by Proteomic Analysis of Patient Dermal Fibroblasts
Source: Biomedicines. 2024 Nov 30;12(12):2749. doi: 10.3390/biomedicines12122749 (PMC11727028; doi:10.3390/biomedicines12122749)
Supplement: Supplementary file 1 [file biomedicines-12-02749-s001.zip › Table S3.pdf]

Supplementary Table S3. GO classification of the 181 DEPs according to PANTHER database

PANTHER protein class	DEPs identified in vEDS dermal fibroblasts
Metabolite interconversion enzyme	PCCB, NDUFB1, **DCXR**, B4GALT7, HACD2, HDHD5, **PPA2**, LPCAT2, MIF, EXT2, XXYLT1, GALNT5, GNPNAT1, MTMR9, CYP1B1, PTDSS1, GALNT10, TPMT, PIK3CA, GSTM3, GGPS1, LDAH, SCO1, **LDHAL6B**, FN3KRP, NNMT, GALE, **ENPP1**, FNTB, SMPD1
RNA metabolism protein	SLC4A1AP, TRA2B, RNPS1, FUS, RPP25L, SMG5, SRSF10, DDX41, POP4, TRA2A, SRSF9, HABP4, NOP10, PNO1, NAA38, LYAR, MBNL2, LARP6, DNAAF10, HNRNPH3, ERI3
Protein-binding activity modulator	RALGPS2, PROCR, RAP2A, **CAP2**, ARL6, RAB22A, **SERPINF1**, USP6NL, TIMP1, RABL3, LXN, **ARHGEF10L**
Scaffold/adaptor protein	CHD2, SPECC1L, ASB1, SNX9, ANKS1B, WDR45, AKAP10, CDC42EP2, PQBP1, SNX2
Protein modifying enzyme	LONP2, ICMT, PTPRF, **CBX4**, TRAPPC12, CDK12, DAP, RNF13, VRK2, AKT3
Transporter	SLC39A10, SLC4A7, ITPR2, **KCNMA1**, CALHM2, SLC39A6, XPO5, SRP19, SLC33A1
Cytoskeletal protein	MYL6B, ACTN1, **LCP1**, **CAP2**, AFDN, GABARAP
Extracellular matrix protein	COL1A1, COL1A2, COL5A1, ELN, FBLN2
Translational protein	GARS1, RPL37, MRPS36, YARS1
Cell adhesion molecule	CNTN3, ITGB3, **ITGA1**
Gene-specific transcriptional regulator	SMAD4, NRBF2, TOX4
Chromatin/chromatin-binding, or -regulatory protein	**CEP135**, HMGN2, CHD2
Membrane traffic protein	DKK1, TMED3, CHMP1B
Intercellular signal molecule	ANGPTL4, DAP
Chaperone	COA3, NPM1
Defense/immunity protein	**ICOSLG**, ANKHD1
DNA metabolism protein	SMARCA4
Calcium-binding protein	**S100A1**
Transfer/carrier protein	TTPAL
Structural protein	ELN
Transmembrane signal receptor	THBD

Proteins with an increased expression are highlighted in bold.
